# Feasibility of a genotyping system for the diagnosis of alpha1 antitrypsin deficiency: a multinational cross-sectional analysis

**DOI:** 10.1186/s12931-022-02074-x

**Published:** 2022-06-10

**Authors:** José Luis Lopez-Campos, Lourdes Osaba, Karen Czischke, José R. Jardim, Mariano Fernandez Acquier, Abraham Ali, Hakan Günen, Noelia Rapun, Estrella Drobnic, Marc Miravitlles

**Affiliations:** 1 Unidad Médico-Quirúrgica de Enfermedades Respiratorias, Instituto de Biomedicina de Sevilla (IBiS), Hospital Universitario Virgen del Rocío, Universidad de Sevilla, Avda. Manuel Siurot, s/n, 41013 Seville, Spain; 2 CIBER de Enfermedades Respiratorias (CIBERES), Instituto de Salud Carlos III, Madrid, Spain; 3 Progenika Biopharma, a Grifols Company, Derio, Vizcaya Spain; 4 Departamento de Neumología, Clínica Alemana de Santiago, Universidad del Desarrollo, Santiago, Chile; 5 Centro de Reabilitação Pulmonar da Escola Paulista de Medicina da Universidade Federal de São Paulo (EPM/Unifesp), São Paulo, Brazil; 6 Servicio de Neumonología, Hospital Cetrángolo, Vicente López, Buenos Aires Argentina; 7 Departamento Médico, Fundación Neumológica Colombiana, Bogotá, D.C., Colombia; 8 Süreyyapaşa Research and Training Center for Chest Diseases and Thoracic Surgery, University of Health Sciences, Istanbul, Turkey; 9 Scientific and Medical Affairs, Grifols, Barcelona, Spain; 10 Servicio de Neumología, Hospital Universitari Vall d’Hebron, Vall d’Hebron Research Institute (VHIR), Vall d’Hebron Barcelona Hospital Campus, Barcelona, Spain

**Keywords:** Alpha1 antitrypsin deficiency, Diagnosis, Buccal swab, Dried blood spots, Genotyping

## Abstract

**Introduction:**

Currently, strategies for improving alpha1 antitrypsin deficiency (AATD) diagnosis are needed. Here we report the performance of a multinational multiplex-based genotyping test on dried blood spots and buccal swabs sent by post or courier and with web registration for subjects with suspected AATD in Argentina, Brazil, Chile, Colombia, Spain, and Turkey.

**Methods:**

This was an observational, cross-sectional analysis of samples from patients with suspected AATD from March 2018 to January 2022. Samples were coded on a web platform and sent by post or courier to the central laboratory in Northern Spain. Allele-specific genotyping for the 14 most common mutations was carried out with the A1AT Genotyping Test (Progenika-Grifols, Spain). SERPINA1 gene sequencing was performed if none of the mutations were found or one variant was detected in heterozygous status and the AAT serum level was < 60 mg/dl, or if requested by the clinician in charge.

**Results:**

The study included 30,827 samples: 30,458 (94.7%) with final results after direct genotyping and 369 (1.1%) with additional gene sequencing. Only 0.3% of the samples were not processed due to their poor quality. The prevalence of the most frequent allele combinations was MS 14.7%, MZ 8.6%, SS 1.9%, SZ 1.9%, and ZZ 0.9%. Additionally, 70 cases with new mutations were identified. Family screening was conducted in 2.5% of the samples. Samples from patients with respiratory diseases other than COPD, including poorly controlled asthma or bronchiectasis, also presented AATD mutations.

**Conclusions:**

Our results confirm the viability of this diagnostic system for genotyping AATD conducted simultaneously in different countries. The system has proved satisfactory and can improve the timely diagnosis of AATD.

**Supplementary Information:**

The online version contains supplementary material available at 10.1186/s12931-022-02074-x.

## Introduction

Despite the proven importance of case identification, early diagnosis of alpha1 antitrypsin deficiency (AATD) currently continues to be a challenge for clinicians and the health system [1]. The reasons for this lack of diagnosis are multifactorial and are related to the lack of systematic screening of potential patients and the need for a quick, simple method to confirm highly suspicious cases, among others [2–4].

In Spain, a new national circuit for diagnosing AATD was established in 2018. Coordinated by the Spanish Network of AATD (REDAAT), it is based on the genetic analysis of dried blood spots (DBS) or buccal swab sampling by a central laboratory (Progenika Biopharma, Derio, Vizcaya, Spain) using the A1AT Genotyping Test that identifies the 14 most frequent deficient variants simultaneously. Our previous analysis showed that the availability of this diagnostic procedure with an easy, convenient sample delivery system and web registration has proved useful at a national level [5]. Interestingly, these positive results could have been influenced by the fact that all the samples originated from Spain, with faster local delivery services. However, since then, the system has been implemented in various Latin American and European countries, with different health systems and postal services, and the number of samples received by the central laboratory from different parts of the world has also risen considerably. It is therefore necessary to demonstrate the viability of the Progenika diagnostic system at a global level.

In this study, we aimed to evaluate the performance of a multinational diagnostic procedure consisting of a multiplex-based genotyping test on samples sent by mail and with web registration on DBS and buccal swabs for subjects with suspected AATD simultaneously received from Argentina, Brazil, Chile, Colombia, Spain, and Turkey. The results will show whether this diagnostic system with long-distance postal delivery is a reliable system for the study of AATD at a more global level.

## Methods

This was an observational, cross-sectional analysis analyzing the anonymized data included on the Progenika web platform (https://grifolsalpha1test.com/) from March 12^th^, 2018, to January 10^th^, 2022. The diagnostic kits for sampling with the DBS and buccal swab were provided to participating centers free of charge by Grifols (Barcelona, Spain) upon request from the treating physicians. For the current analysis, all the samples from Argentina, Brazil, Chile, Colombia, Spain, and Turkey, were analyzed. The methodology has already been described [5]. Briefly, the samples were registered on the web platform through a unique code individually associated with each kit and sent by post to the reference laboratory at the Progenika headquarters in Vizcaya, Northern Spain. In Spain, each sample was sent by post individually. For those geographical areas outside Spain, a transport company collected the samples and periodically sent them to the central laboratories.

When registering the sample on the website, clinicians were asked to include some clinical data about the patient that included age, current smoking status (smoker, former smoker or never smoker), serum AAT level, and forced expiratory volume in one second (FEV_1_) expressed as a percentage of its predicted value, as well as the reasons, chosen from a list, for requesting the test. These data were not compulsory to register the sample. For this analysis, AAT levels were categorized in 4 groups as follows: values below 60 mg/dl, between 60 and 90 mg/dl, between 90 and 120 mg/dl and above 120 mg/dl. Lost samples were considered to be those that at the time of this analysis had not been received in the laboratory and more than 60 days had passed since their online registration, or those with recording errors on the website.

All cases with suspected AATD were considered for inclusion in the diagnostic procedure. These included cases with internationally acknowledged reasons for studying AATD, i.e., Chronic Obstructive Pulmonary Disease (COPD), poorly controlled asthma, blood relatives of individuals with AATD, bronchiectasis, hepatopathy of unknown etiology, shortness of breath and chronic cough in many family members, decrease in alpha-1 peak in proteinogram, and panniculitis or multiorgan vasculitis of unknown cause. Additionally, for the present analysis two additional indications were also recorded: spouses of individuals with AATD and SARS-CoV-2 infection.

### Genetic testing

Allele-specific genotyping was carried out with the Progenika A1AT Genotyping Test. The test uses polymerase chain reaction amplification to obtain large amounts of the target sequences in the SERPINA1 gene and the Luminex^®^ 200 system to detect previously-labeled amplified fragments, as previously described [5]. The test and OCR100 buccal swab used to collect the samples are CE marked (European Conformity) and United States Food and Drug Administration approved. The test is intended for use with genomic DNA extracted from human whole blood samples collected in K3-ethylenediaminetetraacetic acid (EDTA) tubes, as dried blood spots (DBS), or from human buccal swab samples.

The test can identify the 14 most frequent deficiency variants of the SERPINA1 gene, namely PI*S, PI*Z, PI*I, PI*M procida, PI*M malton, PI*S iiyama, PI*Q0 granite falls, PI*Q0 west, PI*Q0 bellingham, PI*F, PI*P Lowell, PI*Q0 mattawa, PI*Q0 clayton, and PI*M heerlen (see Additional file 1: Table S1). When none of the 14 alleles studied was found, the result was noted as negative and interpreted as an M allele, since the absence of any of these 14 alleles suggests with over 99% probability that the genotype corresponds to PI*M.

### Gene sequencing

SERPINA1 gene sequencing was conducted when none of the 14 mutations were found or when one variant was detected in heterozygous status and the AAT serum level was < 60 mg/dl, or when requested by the clinician in charge [5].

### Ethics

The present study complies with the requirements of the Helsinki declaration for studies with human beings. The patients signed a written informed consent authorizing the clinicians to carry out the genetic study according to each national legislation. The personal data of the patients were kept under strict confidentiality in compliance with the provisions of Organic Law 3/2018, of December 5, Protection of Personal Data and Guarantee of digital rights (LOPDGDD) and its development regulations, and in accordance with the provisions of Regulation (EU) 2016/679 of the European Parliament and of the Council of April 27, 2016 regarding the protection of natural persons with regard to the processing of personal data and the free circulation of these data. Additionally, the sampling and shipment complied with all regulations from the different participating countries. The biological samples related to the study were numbered with a code to guarantee the confidentiality of the sample and the associated clinical data. The relationship between this code and the medical record number was kept by the clinician under their sole responsibility and custody. To ensure confidentiality of the information, a different company (Haiko Technologies, Bilbao, Spain) acted as an intermediary between Progenika and Grifols. There was therefore no data in the database that could be used to identify the patients.

### Statistics

Statistical analyses were performed with IBS SPSS Statistics (IBM Corporation, Armonk, NY), version 28.0. Data were described using absolute counts with relative frequencies in parentheses for categorical variables. A hit rate was defined as the percentage of severe genotypes in each geographical area. Additionally, a testing/population ratio was also estimated. Quantitative data were summarized with the mean and standard deviation (SD) in parentheses. Maps were created using a Microsoft Excel (Microsoft Corporation, Redmond, WA) spreadsheet.

## Results

**Fig. 1:**
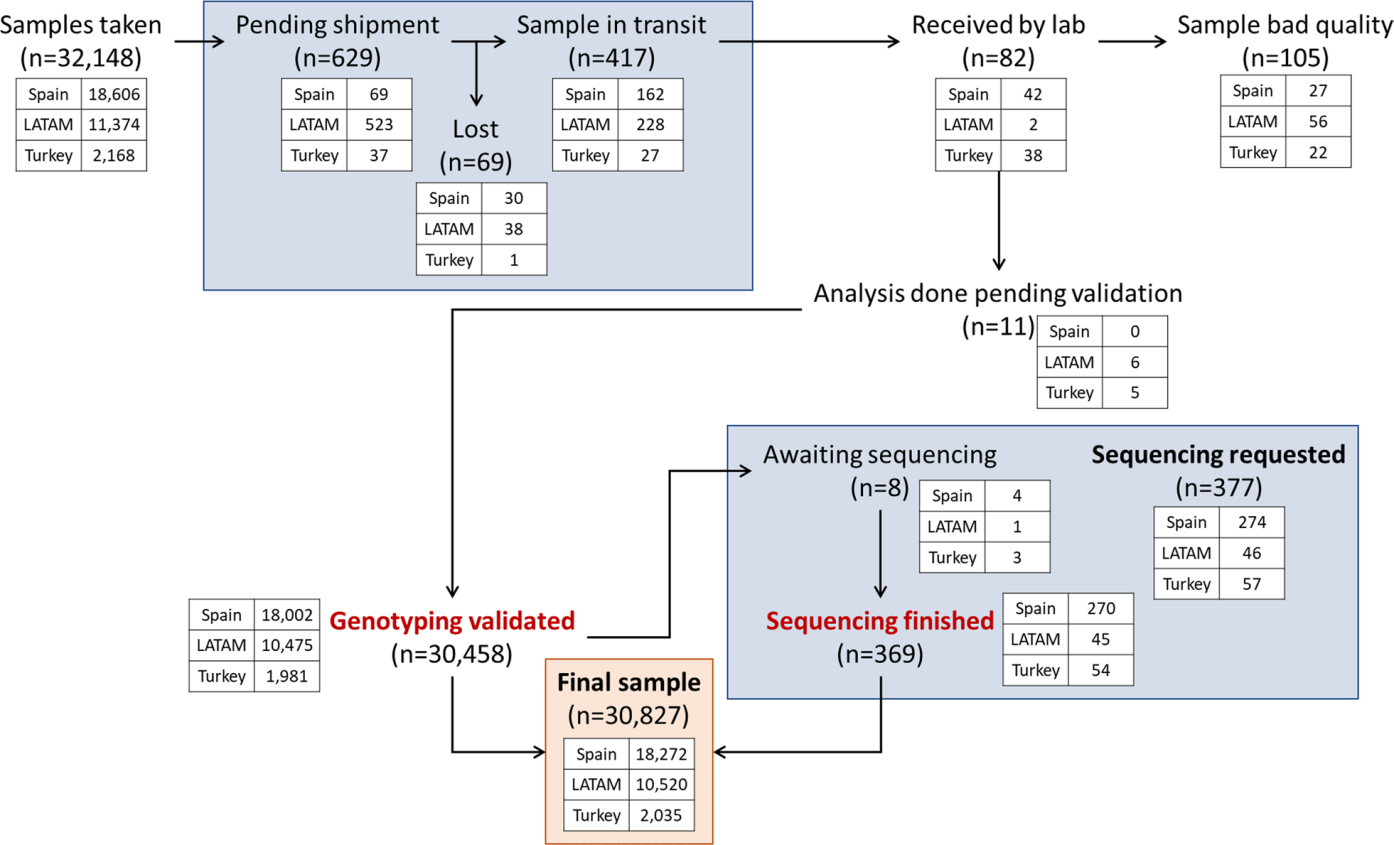
Flow chart of the current distribution of the cases in the different steps of the procedure. Results expressed in absolute numbers in total and by geographical area. The Progenika system is a live circuit currently in use, so there are samples in all steps of the procedure

During the study period, there were 32,148 samples either recorded in the system or received in the laboratory, of which 30,827 (95.8%) had been processed at the time of the present report: 30,458 (94.7%) with final results after direct genotyping and 369 (1.1%) with gene sequencing (Fig. 1). One hundred and five samples (0.3%) were not processed due to the poor quality of the sample and 69 (0.2%) did not arrive at the laboratory, corresponding to recording errors or sample losses during shipment. Over time, there was a progressive increase in the inclusion of patients, but with a pronounced impact due to the coronavirus pandemic (see Additional file 1: Figs. S1 and S2).

**Fig. 2:**
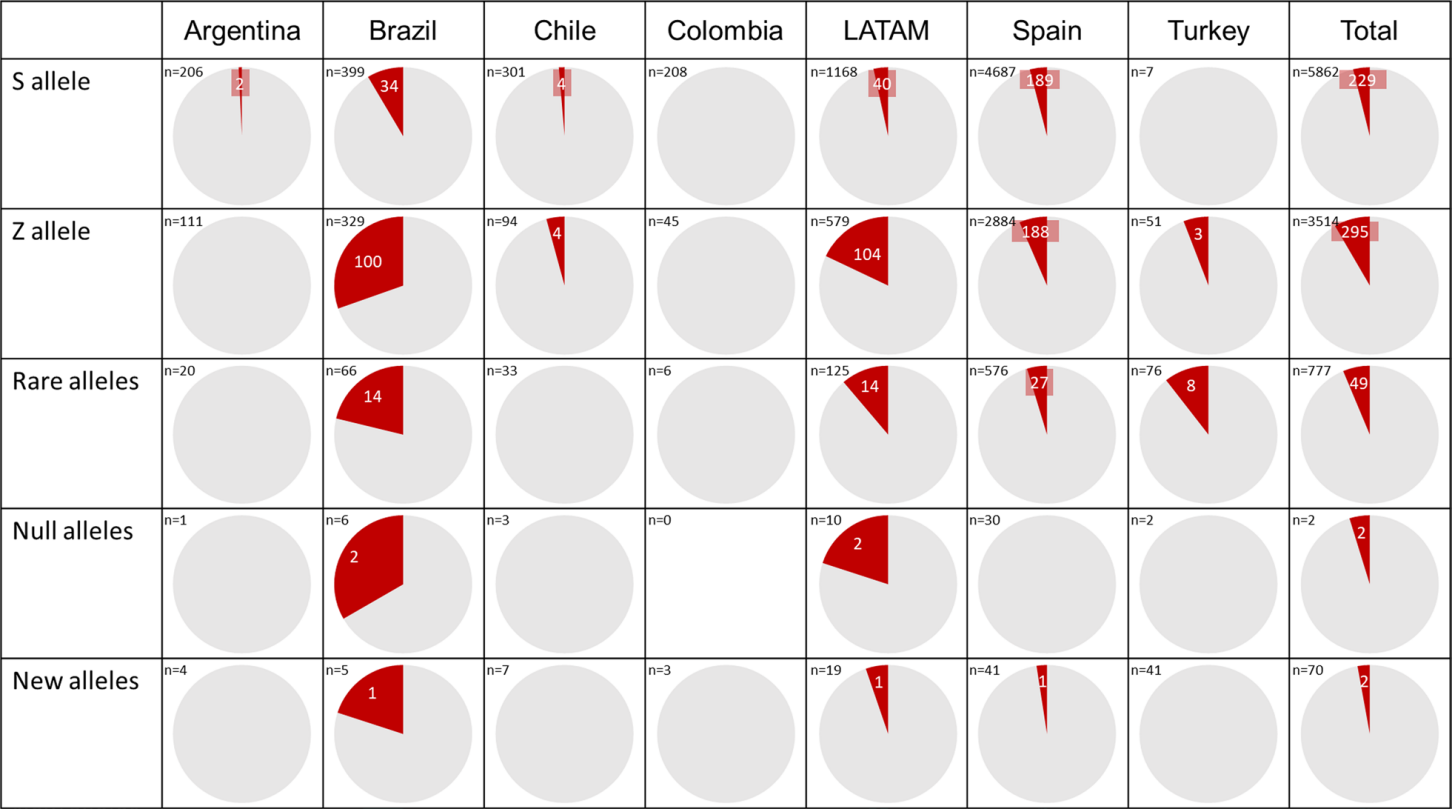
Number of cases detected by familiar screening in each different location. Red: cases detected by familiar screening. Grey: index cases

The subjects studied included 6614 (21.5%) current smokers, 12,495 (40.5%) ex-smokers, and 11,718 (38.0%) never smokers (see Table 1), and their mean age was 56.1 (SD: 18.0) years. FEV_1_% predicted was available for 15,247 (49.5%) cases, with a mean value of 70.2 (SD: 24.4) %. AAT serum levels were available in 5,685 (18.4%) cases, with a mean value of 93.5 (SD: 34.4) mg/dl. The distribution of cases according to the different AAT cut-off values was: 1275 (22.4%) were above 120 mg/dl, 1714 (30.1%) were between 90 and 120 mg/dl, 1970 (34.7%) were between 60 and 89 mg/dl, and 726 (12.8%) were below 60 mg/dl. Of the samples, 30,063 (97.5%) were index cases (see Fig. 2). The majority of samples, 24,114 (78.2%) were from buccal swabs. The distribution of the different sample types between countries is shown in Additional file 1: Fig. S3.

**Table 1:** Characteristics of the cases included

	Argentina (n = 2491)	Brazil (n = 2620)	Chile (n = 3352)	Colombia (n = 2057)	LATAM (n = 10,520)	Spain (n = 18,272)	Turkey (n = 2035)	Total (n = 30,827)
Age (year)	55.0 (16.7)	59.1 (17.0)	55.7 (19.9)	68.7 (13.1)	58.9 (17.9)	54.5 (18.1)	56.0 (15.1)	56.1 (18.0)
Smoking habits								
Current smokers	583 (23.4)	338 (12.9)	665 (19.8)	90 (4.4)	1676 (15.9)	4307 (23.6)	631 (31.0)	6614 (21.5)
Ex-smokers	1014 (40.7)	1509 (57.6)	1143 (34.1)	931 (45.3)	4597 (43.7)	6959 (38.1)	939 (46.1)	12,495 (40.5)
Never smokers	894 (35.9)	773 (29.5)	1544 (46.1)	1036 (50.4)	4247 (40.4)	7006 (38.3)	465 (22.9)	11,719 (38.0)
Alpha1 antitrypsin available	62 (2.5)	270 (10.3)	96 (2.9)	11 (0.5)	439 (4.2)	5137 (28.1)	109 (5.4)	5685 (18.4)
Alpha1 antitrypsin (mg/dl)	77.1 (49.1)	95.7 (48.8)	94.3 (48.0)	57.8 (57.5)	91.8 (49.4)	94.1 (32.0)	74.6 (55.7)	93.5 (34.4)
FEV1 available	1834 (73.6)	1603 (61.2)	807 (24.1)	0 (0)	4244 (40.3)	10,612 (58.1)	391 (19.2)	15,247 (49.5)
FEV1 (%)	60.7 (17.3)	51.5 (21.5)	65.4 (22.8)	–	58.1 (20.8)	75.6 (23.9)	53.7 (18.8)	70.2 (24.4)

Data expressed as mean (standard deviation) or as absolute (relative) frequencies depending on the nature of the variable

The reasons to include the patient in the diagnostic procedure are summarized in Table 2. The most frequent reason was COPD, followed by poorly controlled asthma, and bronchiectasis. In total, 25% of the samples did not report any specific reason. Interestingly, only 2.5% of the samples were conducted as part of family screening. There was a considerable variability between countries. Samples in Latin American (LATAM) countries and Turkey were more frequently taken for COPD, whereas in Spain there was a wider distribution of the reasons behind AATD testing. Individuals with respiratory diseases other than COPD, including poorly controlled asthma or bronchiectasis, also presented AATD mutations.

**Table 2:** Reasons to include the patient in the system

	Argentina (n = 2491)	Brazil (n = 2620)	Chile (n = 3352)	Colombia (n = 2057)	LATAM (n = 10,520)	Spain (n = 18,272)	Turkey (n = 2035)	Total (n = 30,827)
COPD	1234 (49.5)	1909 (72.9)	1189 (35.5)	1936 (94.1)	6268 (59.6)	7025 (38.4)	1936 (95.1)	15,230 (49.4)
Poorly controlled asthma	682 (27.4)	183 (7.0)	325 (9.7)	61 (3.0)	1251 (11.9)	2095 (11.5)	35 (1.7)	3381 (11.0)
Bronchiectasis	127 (5.1)	267 (10.2)	155 (4.6)	76 (3.7)	625 (5.9)	777 (4.3)	33 (1.6)	1435 (4.7)
Serum AAT concentration level below the limit of normality	0 (0.0)	34 (1.3)	2 (0.1)	0 (0.0)	36 (0.3)	321 (1.8)	0 (0.0)	357 (1.2)
Blood relatives of individuals with AATD	2 (0.1)	176 (6.7)	11 (0.3)	0 (0.0)	189 (1.8)	548 (3.0)	28 (1.4)	765 (2.5)
Spouses of individuals with AATD	0 (0.0)	8 (0.3)	1 (0.0)	0 (0.0)	9 (0.1)	21 (0.1)	0 (0.0)	30 (0.1)
Hepatopathy of unknown cause	6 (0.2)	79 (3.0)	6 (0.2)	0 (0.0)	91 (0.9)	200 (1.1)	3 (0.1)	294 (1.0)
Dyspnea and chronic cough in many family members	272 (10.9)	39 (1.5)	93 (2.8)	0 (0.0)	404 (3.8)	368 (2.0)	7 (0.3)	779 (2.5)
Decreased or lack of peak alpha-1 protein on proteinogram	3 (0.1)	17 (0.6)	3 (0.1)	1 (0.0)	24 (0.2)	551 (3.0)	2 (0.1)	577 (1.9)
Panniculitis or multiorgan vasculitis of unknown cause	8 (0.3)	14 (0.5)	2 (0.1)	0 (0.0)	24 (0.2)	16 (0.1)	1 (0.0)	41 (0.1)
SARS-CoV-2 infection	4 (0.2)	28 (1.1)	190 (5.7)	0 (0.0)	222 (2.1)	1262 (6.9)	0 (0.0)	1484 (4.8)
None of the above	191 (7.7)	85 (3.2)	1453 (43.3)	27 (1.3)	1756 (16.7)	5937 (32.5)	8 (0.4)	7701 (25.0)

Data expressed as absolute (relative) frequencies. Percentages referred to the total number per country

The average number of days taken for the different procedural steps is shown in the Additional file 1: Fig. S4. While the samples were genotyped when received at the laboratory, sequencing required there to be a minimum number of samples in order to be processed together. Consequently, sequencing times were longer. Gene sequencing was carried out in 369 (1.2%) cases (Additional file 1: Table S2). In all cases, the sequencing results were consistent with the A1AT Genotyping Test results. In 94 (25.4%) cases, sequencing revealed additional mutations.

**Fig. 3:**
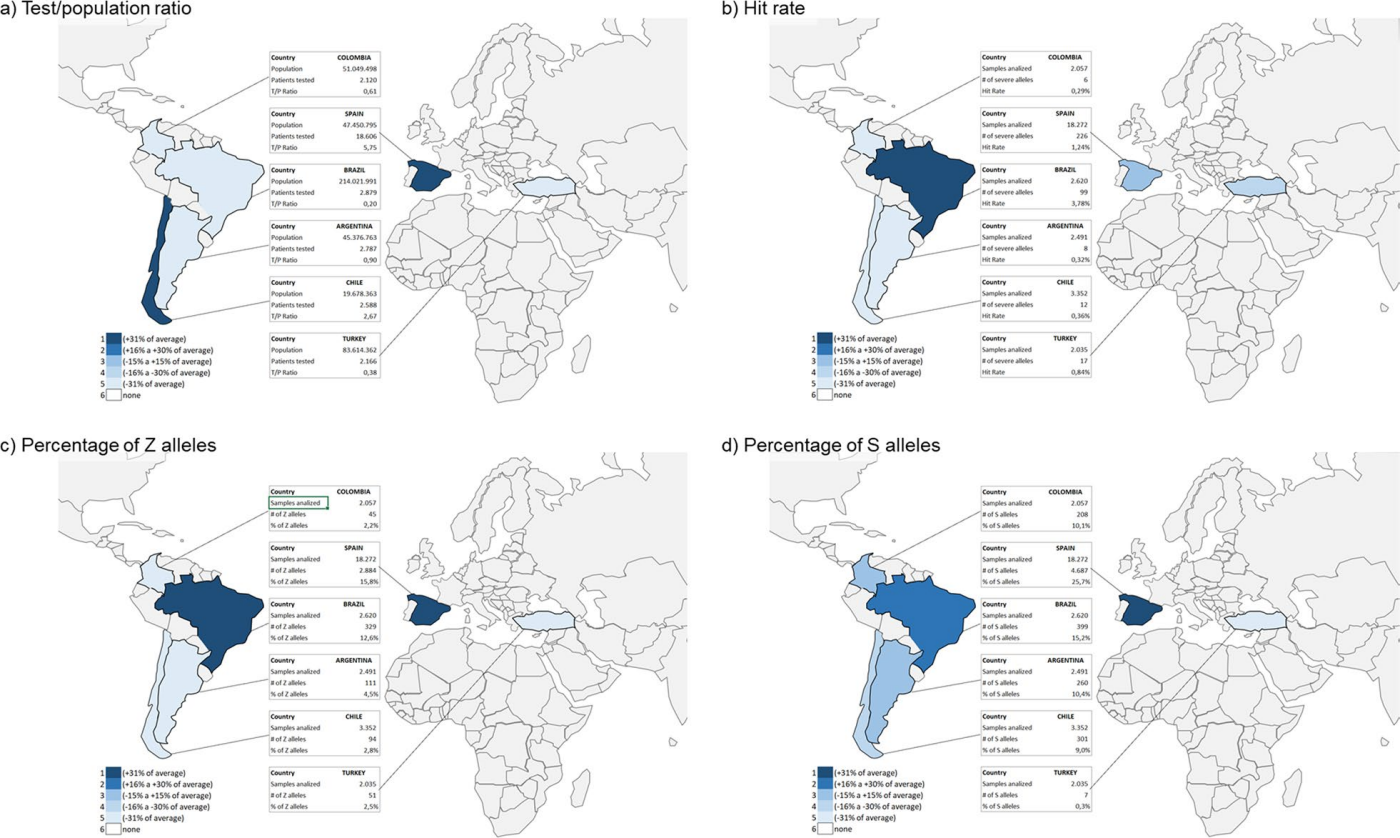
World map showing the ratio testing/population, the hit rate and the percentage of Z and S alleles. This is not an accurately scaled map

**Fig. 4:**
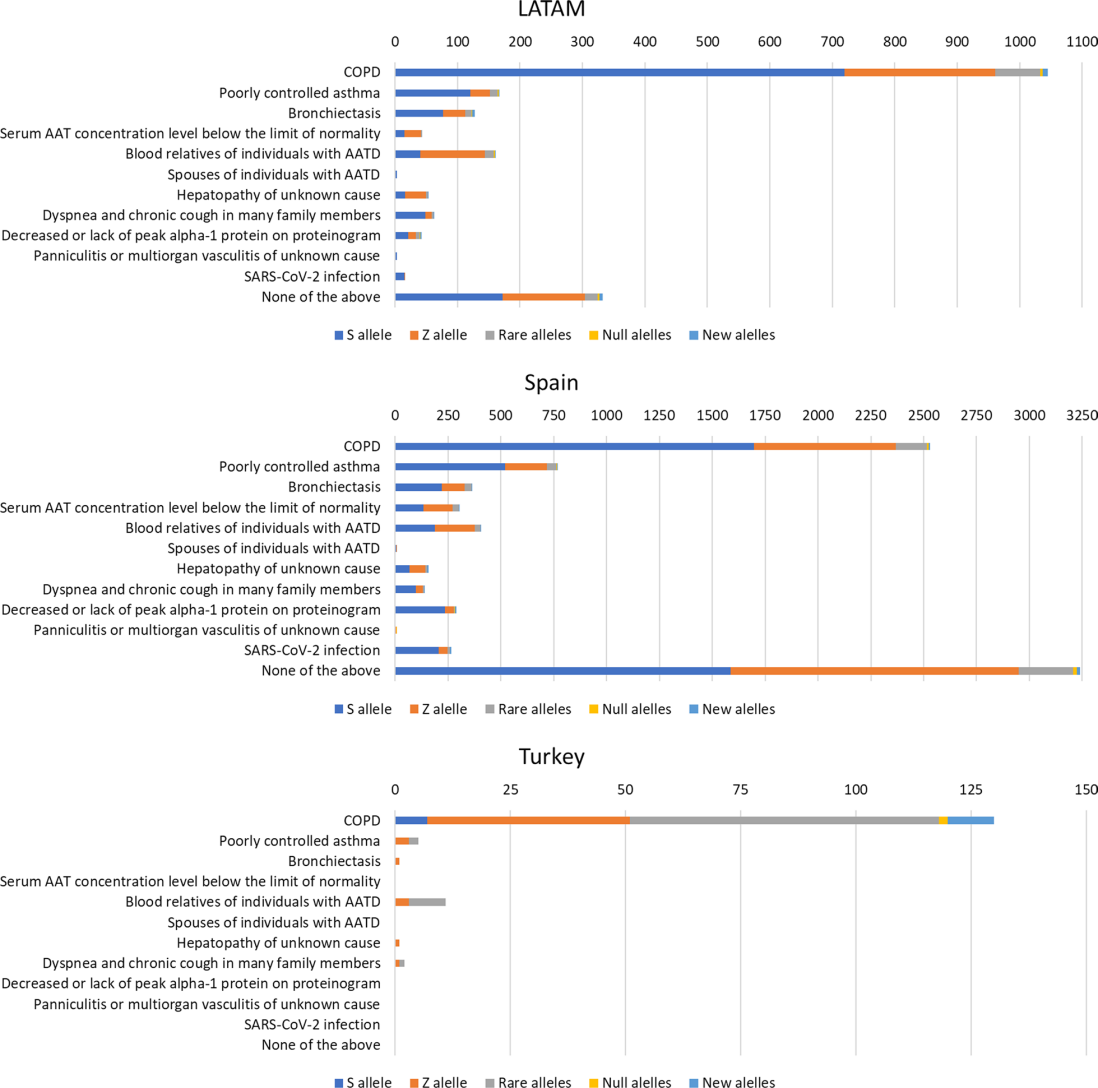
Allele distribution according to the different genotyping reasons

The complete list and distribution of mutations is shown in Table 3. Altogether, there were 9,528 (30.9%) samples carrying at least one mutation, with differences between regions. In Turkey, there was a small percentage of S allele, with a predominance of rare alleles and an increase in new mutations compared to other areas. The most frequent rare alleles in Turkey were: Plowell in 36 cases (25.7% of the mutations found), Mmalton in 24 cases (17.1% of the mutations found), and allele I in 13 (9.2% of the mutations found). In contrast, the S allele was predominant in Spain and Latin American countries, followed by the Z allele. Index cases showed a different distribution among the different participating areas (Fig. 2). The percentage distribution of the S and Z alleles, the testing/population ratio and the hit rate between the different countries are shown in Fig. 3. The distribution of the different alleles according to the reason for studying the genotype is shown in Fig. 4 (Additional file 1: Fig. S5 for the different Latin American countries) and Table 4. Although the majority of cases (49.4%) were patients with COPD, other clinical conditions also carried mutations. Specifically, 3,381 cases (11.0%) were poorly controlled asthma and 1,435 (4.7%) were patients with bronchiectasis.

**Table 3:** Description of the results of the genotypes found

	Argentina (n = 2491)	Brazil (n = 2620)	Chile (n = 3352)	Colombia (n = 2057)	LATAM (n = 10,520)	Spain (n = 18,272)	Turkey (n = 2035)	Total (n = 30,827)
No mutation	2107 (84.6)	1875 (71.6)	2929 (87.4)	1800 (87.5)	8711 (82.8)	10,693 (58.5)	1895 (93.1)	21,300 (69.1)
Any mutation	384 (15.4)	745 (28.4)	423 (12.6)	257 (12.5)	1809 (17.2)	7579 (41.5)	140 (6.9)	9528 (30.9)
Frequent genotypes:	359 (14.4; 93.5)	670 (25.6; 89.9)	381 (11.4; 90.1)	248 (12.1; 96.5)	1658 (15.8; 91.7)	6934 (37.9; 91.5)	53 (2.6; 37.9)	8645 (28.0; 90.7)
MS	237 (9.5; 61.7)	332 (12.7; 44.6)	287 (8.6; 67.8)	193 (9.4; 75.1)	1049 (10.0; 58.0)	3466 (19.0; 45.7)	7 (0.3; 5.0)	4522 (14.7; 47.5)
MZ	95 (3.8; 24.7)	194 (7.4; 26.0)	76 (2.3; 18.0)	35 (1.7; 13.6)	400 (3.8; 22.1)	2200 (12.0; 29.0)	39 (1.9; 27.9)	2639 (8.6; 27.7)
SS	12 (0.5; 3.1)	22 (0.8; 3.0)	8 (0.2; 1.9)	8 (0.4; 3.1)	50 (0.5; 2.8)	550 (3.0; 7.3)	0 (0.0; 0.0)	600 (1.9; 6.3)
SZ	10 (0.4; 2.6)	43 (1.6; 5.8)	6 (0.2; 1.4)	6 (0.3; 2.3)	65 (0.6; 3.6)	536 (2.9; 7.1)	0 (0.0; 0.0)	601 (1.9; 6.3)
ZZ	5 (0.2; 1.3)	79 (3.0; 10.6)	4 (0.1; 0.9)	6 (0.3; 2.3)	94 (0.9; 5.2)	181 (1.0; 2.4)	7 (0.3; 5.0)	282 (0.9; 3.0)
Allele S	260 (10.4; 67.7)	399 (15.2; 53.6)	301 (9.0; 71.2)	208 (10.1; 80.9)	1168 (11.1; 64.6)	4687 (25.7; 61.8)	7 (0.3; 5.0)	5862 (19.0; 61.5)
Allele Z	111 (4.5; 28.9)	329 (12.6; 44.2)	94 (2.8; 22.2)	45 (2.2; 17.5)	579 (5.5; 32.0)	2884 (15.8; 38.1)	51 (2.5; 36.4)	3514 (11.4; 36.9)
Rare alleles	20 (0.8; 5.2)	66 (2.5; 8.9)	33 (1.0; 7.8)	6 (0.3; 2.3)	125 (1.2; 6.9)	576 (3.2; 7.6)	76 (3.7; 54.6)	777 (2.5; 8.2)
Null alleles	1 (0.0; 0.3)	6 (0.2; 0.8)	3 (0.1; 0.7)	0 (0.0; 0.0)	10 (0.1; 0.6)	31 (0.2; 0.4)	2 (0.1; 1.4)	43 (0.1; 0.4)
New alleles	4 (0.2; 1.0)	5 (0.2; 0.7)	7 (0.2; 1.7)	3 (0.1; 1.2)	19 (0.2; 1.1)	42 (0.2; 0.5)	10 (0.5; 7.1)	71 (0.2; 0.7)

Data expressed as absolute numbers with percentages in parenthesis; first value showing percentages referred to the total number of samples in the geographical area, second value showing percentages referred to the total number of cases with mutations in the geographical area

**Table 4:** Distribution of frequent genotypes according to the background disease an geographical areas

Diseases	Frequent genotypes	Argentina	Brazil	Chile	Colombia	LATAM	Spain	Turkey	Total
COPD	n	1234	1909	1189	1936	6268	7025	1936	15,229
	MS	126 (10.2)	249 (13.0)	100 (8.4)	185 (9.6)	660 (10.5)	1337 (19.0)	7 (0.4)	2004 (13.2)
	MZ	35 (2.8)	83 (4.3)	13 (1.1)	13 (1.1)	153 (2.4)	483 (6.9)	34 (1.8)	670 (4.4)
	SS	7 (0.6)	15 (0.8)	2 (0.2)	2 (0.2)	32 (0.5)	180 (2.6)	0 (0)	212 (1.4)
	SZ	5 (0.4)	13 (0.7)	1 (0.1)	1 (0.1)	24 (0.4)	140 (2.0)	0 (0)	164 (1.1)
	ZZ	4 (0.3)	42 (2.2)	2 (0.2)	2 (0.1)	52 (0.8)	74 (1.1)	6 (0.3)	132 (0.9)
Poorly controlled asthma	n	682	183	325	61	1251	2095	35	3381
	MS	62 (9.1)	20 (10.9)	28 (8.6)	5 (8.2)	115 (9.2)	409 (19.5)	0 (0)	524 (15.5)
	MZ	15 (2.2)	9 (4.9)	4 (1.2)	1 (1.6)	29 (2.3)	155 (7.4)	1 (2.9)	185 (5.5)
	SS	2 (0.3)	2 (1.1)	1 (0.3)	0 (0)	5 (0.4)	65 (3.1)	0 (0)	70 (2.1)
	SZ	0 (0)	0 (0)	0 (0)	0 (0)	0 (0)	37 (1.8)	0 (0)	37 (1.1)
	ZZ	0 (0)	1 (0.5)	0 (0)	1 (1.6)	2 (0.2)	19 (0.9)	1 (2.9)	22 (0.7)
Bronchiectasis	n	127	267	155	76	625	777	33	1435
	MS	5 (3.9)	39 (14.6)	16 (10.3)	7 (9.2)	67 (10.7)	154 (19.8)	0 (0)	221 (15.4)
	MZ	7 (5.5)	11 (4.1)	2 (1.3)	0 (0)	20 (3.2)	81 (10.4)	1 (3.0)	102 (7.1)
	SS	0 (0)	5 (1.9)	1 (0.6)	0 (0)	6 (1.0)	36 (4.6)	0 (0)	42 (2.9)
	SZ	0 (0)	4 (1.5)	0 (0)	0 (0)	4 (0.6)	22 (2.8)	0 (0)	26 (1.8)
	ZZ	0 (0)	9 (3.4)	0 (0)	0 (0)	9 (1.4)	18 (2.3)	0 (0)	27 (1.9)
Panniculitis or multiorgan vasculitis of unknown cause	n	8	14	0	0	22	16	1	41
	MS	1 (12.5)	2 (14.3)	0 (0)	0 (0)	3 (12.5)	3 (18.8)	0 (0)	6 (14.6)
	MZ	0 (0)	0 (0)	0 (0)	0 (0)	0 (0)	1 (6.3)	0 (0)	1 (2.4)
	SS	0 (0)	0 (0)	0 (0)	0 (0)	0 (0)	0 (0)	0 (0)	0 (0)
	SZ	0 (0)	0 (0)	0 (0)	0 (0)	0 (0)	0 (0)	0 (0)	0 (0)
	ZZ	0 (0)	0 (0)	0 (0)	0 (0)	0 (0)	0 (0)	0 (0)	0 (0)

Data expressed as absolute numbers with percentages in parenthesis; showing percentages referred to the total number of samples in the geographical area with that particular disease

## Discussion

This report summarizes the evaluation of the Progenika diagnostic procedure for patients with suspected AATD in different countries. The diagnostic system allows for the simultaneous testing of 14 genetic variants using either DBS or buccal swabs utilizing online registration and samples sent by post. Our results confirm that this diagnostic circuit is reliable, with most samples arriving at the reference laboratory in good condition to allow for genotyping.

The diagnosis of AATD continues to be a challenge for clinicians due to the low degree of suspicion and the lack of availability of rapid, simple circuits that permit more universal access to genotyping techniques aimed at diagnosing the disease [6–8]. This new diagnostic circuit represents an opportunity to improve the underdiagnosis of this clinical condition. The diagnostic system presents four major advantages. First of all, having a non-invasive sample collection that does not require a specially dedicated or complex infrastructure represents a considerable advantage. Secondly, the nature of the sample and the conservation measures used allow it to be sent by post from each country, without requiring any special security measures. Thirdly, the guaranteed anonymization of data means that it can be aligned with the data protection legislation of the countries that wish to implement it. Finally, the rapid processing of the sample, with online results obtained in real time by the requesting physician, greatly speed up the diagnosis. Here, we have shown that the Progenika laboratory has the capacity to genotype and analyze all the samples in a short period of time, with the possibility of gene sequencing on demand or after unexpected results from both index cases and relatives. Consequently, this is a system that can be implemented both in big reference hospitals and in remote small areas. Of note, this system has been successfully used in other European countries [9, 10]. In fact, the system is so simple that it could well be established as a screening method. The results therefore help us to draw and update a new map of the prevalence of these mutations in all the participating countries, including more isolated areas. Additionally, it can help us to identify new cases and improve registration in international registries [11]. In this regard, In Spain the system is in use since 2018 and therefore the current report provides much more data, simultaneously and faster collected in this country. However, the situation is slightly different in Latin American countries. Here, the system has just been adopted and there was a need to show that this system also works in these countries despite the considerable distance and amount of samples received by one central laboratory, as reported here.

However, there are some considerations to take into account before implementing it in a particular area. One methodological consideration of the circuit we have found is the registration of the samples on the website. Despite the potential advantage of having an online system for data registration, this system depends on the clinician correctly registering the sample code on the website to link the results to the clinical data. Although clinical data are not compulsory, it is of help to have this information. The most important example of this is the availability of AAT levels, since, if they do not match with the genotyping results, the central laboratory continues with the sequencing directly without the intervention of the clinician. On a cautionary note, there is also the context of the current SARS-CoV-2 pandemic. Due to the high transmissibility of this virus, there may be concerns about the safety of taking and sending biological samples by post. Fortunately, the liquid preserving the samples has antiviral properties (ORAcollect fact sheet). In addition, the samples are stored in a hermetically sealed tube in a double protective envelope, which makes the system extremely safe. So far, no cases of COVID19 transmission have been reported due to handling, sending or receiving these samples. Another note of caution should be considered in the cases with hepatopathy of unknown cause. The clinicians participating in this circuit were mostly pulmonologists or general practitioners. Therefore, cases with hepatopathy of unknown cause may be under-represented. The addition of liver disease specialists to the circuit would contribute to the detection of cases of AATD in this clinical context. Another note to consider is that the multiplex system studies the 14 most common mutations. Therefore, identification of the Pi*M is carried out by exclusion, since the absence of any of these 14 alleles suggests with over 99% probability that the genotype corresponds to PI*M. However, in case of a discrepancy with AATD serum levels, or on a clinician’s request, the gene could be sequenced. Interestingly, this situation has only occurred in a small number of cases during our study, which is especially low given that the 14 genotypes studied include over 99% of the deficient variants observed in the world. The prevalence figures here overestimate the real prevalence of AATD, since this is a highly selected population with high suspicion for AATD, and, therefore, this is not a population-based study. Finally, submission of samples is a key step in the process. It is important to bear in mind that the stability of the sample has been studied in tests carried out for 60 days at room temperature with 3 cycles of peaks from – 20 °C to 50 °C (see technical fact sheet). Under these conditions, optimal results have been obtained in genotyping. In our case, the samples had a delivery time considerably less than 60 days, guaranteeing a shipment of good quality samples.

The evolution of sampling has been significantly influenced by the emergence of the pandemic. As can be seen in the Additional file 1 figures, the influence of the pandemic has been evident on a global scale, but the degree of recovery has varied from country to country. In the coming months, it is expected that the situation will return to pre-pandemic values. In Spain, the decision on whether to do a buccal or a DBS sample depended on a consensual decision between the clinician in charge and the patient (Additional file 1: Fig. S3). In Turkey, only DBS was available, while in Argentina and Chile, a transition has been made from the initial DBS to the buccal swab, and currently, all LATAM countries use buccal swab exclusively. Interestingly, In Spain, the use of DBS increased during the worst months of the pandemic (data not shown). Recently, the possibility of self-sampling has been reported with the use of direct-to-consumer genetic testing for AATD. The results show that not only is it possible to make a correct diagnosis with auto-sampling, but also that it was associated with positive changes in behavior [12].

The way different countries have dealt with genotyping results is interesting and varies widely. In the case of Turkey, the proportion of the S allele is considerably lower: in fact, it is known that the S allele must have arisen in or around the Iberian Peninsula [13]. Accordingly, the distribution of S and Z alleles in Spain are in line with the reported European prevalence of S and Z alleles where there is an increase of the S allele in Spain and Portugal compared to other European countries [13–16]. Due to the historical influence of Europeans from Spain and Portugal on Latin America and the bidirectional migratory movements between Latin America and Europe during centuries, it is to be expected that this S allele is also prevalent in both Spain and Latin America. However, historically, the relationship with Turkey has not been so close, which has a direct consequence on the lower prevalence of this allele. However, the prevalence of rare alleles and new mutations is on the rise in Turkey. These data are interesting as they reveal that mutations in the SERPINA1 gene may be frequent [17]. Therefore, a new mutation could appear and steadily increase in frequency in specific geographic areas over the generations, and this justifies the screening of suspected patients in any location.

Unfortunately, family screening is rarely performed, and in some countries not at all. Relatives are expected to present a higher proportion of mutations, being a highly selected population. However, Brazil seems to be the country with the greatest increase in the number of samples coming from family screening, which may account for the fact that it is the country with the highest percentage of subjects in which a mutation has been identified. Familiar screening provides us with the opportunity to provide genetic education for families and to avoid risk factors in those cases carrying mutations [16].

Interestingly, the prevalence data for mutations differ between geographic areas. In Spain, the number of patients with a mutation of any type is similar to that described in a previous analysis with this same program [5] and lower than in other Northern European countries, for example Poland (87.6%), Germany (62.9%) or Ireland (72.8–85.1%) [18–20], although the frequencies found could be altered by the differences in the populations under study. However, according to our data, this frequency decreases considerably in Latin American countries, with around 17.2% of the samples, and even more so in Turkey, which is below 10%. Since the origin of the two most prevalent mutations (S and Z) were outside Latin America and Turkey, prevalence in these two areas is also expected to be below the European reference. Our current analysis updates the distribution of S and Z alleles in the Spanish population [14, 15, 21] and gives new data on other countries in the world. The increase in P lowell and M malton cases in Turkey is an unexpected finding. P lowell was first described in the US [22]. Although alleles I and P lowell were originally defined at the protein level through isoelectric focusing, they were later characterized at the DNA level [23]. With the current data, we find it difficult to account for such a marked increase in these alleles for this area.

In addition, it is important to note that AATD was detected in conditions other than COPD, including asthma and bronchiectasis as the other most prevalent airway diseases [24–27]. The question arises here about whether the presence of AAT mutations in these populations is simply an epidemiological coincidence, as part of the wider distribution of some of these genotypes in the population [28, 29] or if it has a pathogenetic effect [30, 31]. Interestingly, in the case of bronchiectasis, the existence of mutations described in this group seems to point to cases with unique characteristics [32–35]. Accordingly, some authors suggest screening for AATD in adult patients with non-fully reversible asthma [36]. Additionally, current bronchiectasis guidelines recommend AATD screening in this population [37]. Of note, the number of cases in which the reasons for studying suspected AATD were not on the list of possible causes was higher in Chile and Spain, as shown in Table 2.

In conclusion, our results confirm in different countries our previous findings in Spain about the good performance of the Progenika system for genotyping AATD conducting simultaneous testing of 14 genetic variants from DBS and buccal swabs, with web registration and sent by post. The system has therefore proved satisfactory and can improve the timely diagnosis of AATD.

## Supplementary Information

**Additional file 1:** Supplementary material.

## Data Availability

All data generated or analyzed during this study are included in this published article and its additional information files.
