# Supplementary material for: Feasibility of a genotyping system for the diagnosis of alpha1 antitrypsin deficiency: a multinational cross-sectional analysis
Source: Respir Res. 2022 Jun 10;23:152. doi: 10.1186/s12931-022-02074-x (PMC9184812; doi:10.1186/s12931-022-02074-x)
Supplement: Supplementary file 1 — Additional file 1: Supplementary material. [file 12931_2022_2074_MOESM1_ESM.docx]

**SUPPLEMENTARY MATERIAL**

**Feasibility of a genotyping system for the diagnosis of alpha1 antitrypsin deficiency in different countries.**

**Authors:** José Luis Lopez-Campos (1,2), Lourdes Osaba (3), Karen Czischke (4), José R. Jardim (5), Mariano Fernandez Acquier (6), Abraham Ali (7), Hakan Günen (8), Noelia Rapun (3), Estrella Drobnic (9), Marc Miravitlles (2,10)

**Institutions:**

1. Unidad Médico-Quirúrgica de Enfermedades Respiratorias. Instituto de Biomedicina de Sevilla (IBiS). Hospital Universitario Virgen del Rocío/Universidad de Sevilla, Spain.
2. CIBER de Enfermedades Respiratorias (CIBERES). Instituto de Salud Carlos III, Madrid, Spain.
3. Progenika Biopharma, a Grifols company. Derio, Vizcaya, Spain
4. Departamento de Neumología, Clínica Alemana de Santiago, Universidad del Desarrollo, Santiago, Chile.
5. Centro de Reabilitação Pulmonar da Escola Paulista de Medicina da Universidade Federal de São Paulo (EPM/Unifesp), São Paulo, Brazil
6. Servicio de Neumonología, Hospital Cetrángolo, Vicente López, Buenos Aires, Argentina
7. Departamento Médico, Fundación Neumológica Colombiana, Bogotá, D.C., Colombia
8. University of Health Sciences, Süreyyapaşa Research and Training Center for Chest Diseases and Thoracic Surgery, Istanbul, Turkey.
9. Scientific & Medical Affairs, Grifols, Barcelona, Spain.
10. Servicio de Neumología. Hospital Universitari Vall d'Hebron, Vall d'Hebron Research Institute, Barcelona, Spain.

**Correspondence:** JL Lopez-Campos. Hospital Universitario Virgen del Rocío. Avda. Manuel Siurot, s/n. 41013 Seville, Spain. Email: [lcampos@separ.es](mailto:lcampos@separ.es)

Figure 1S. Evolution over time of genetic study requests in Spain, LATAM and Turkey.


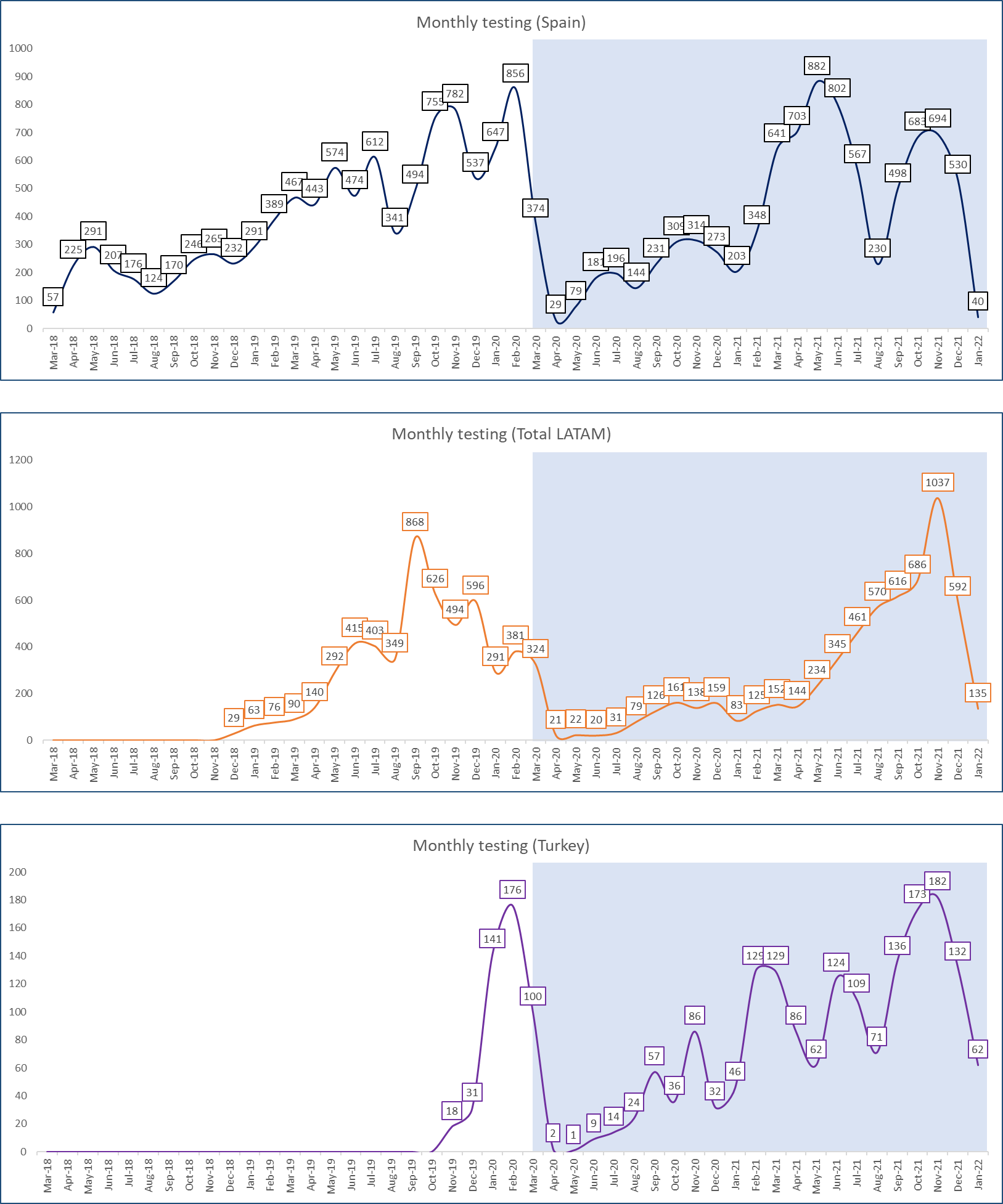


In light blue, effect of Coronavirus pandemic.

Figure 2S. Evolution over time of requests for genetic studies by LATAM countries.


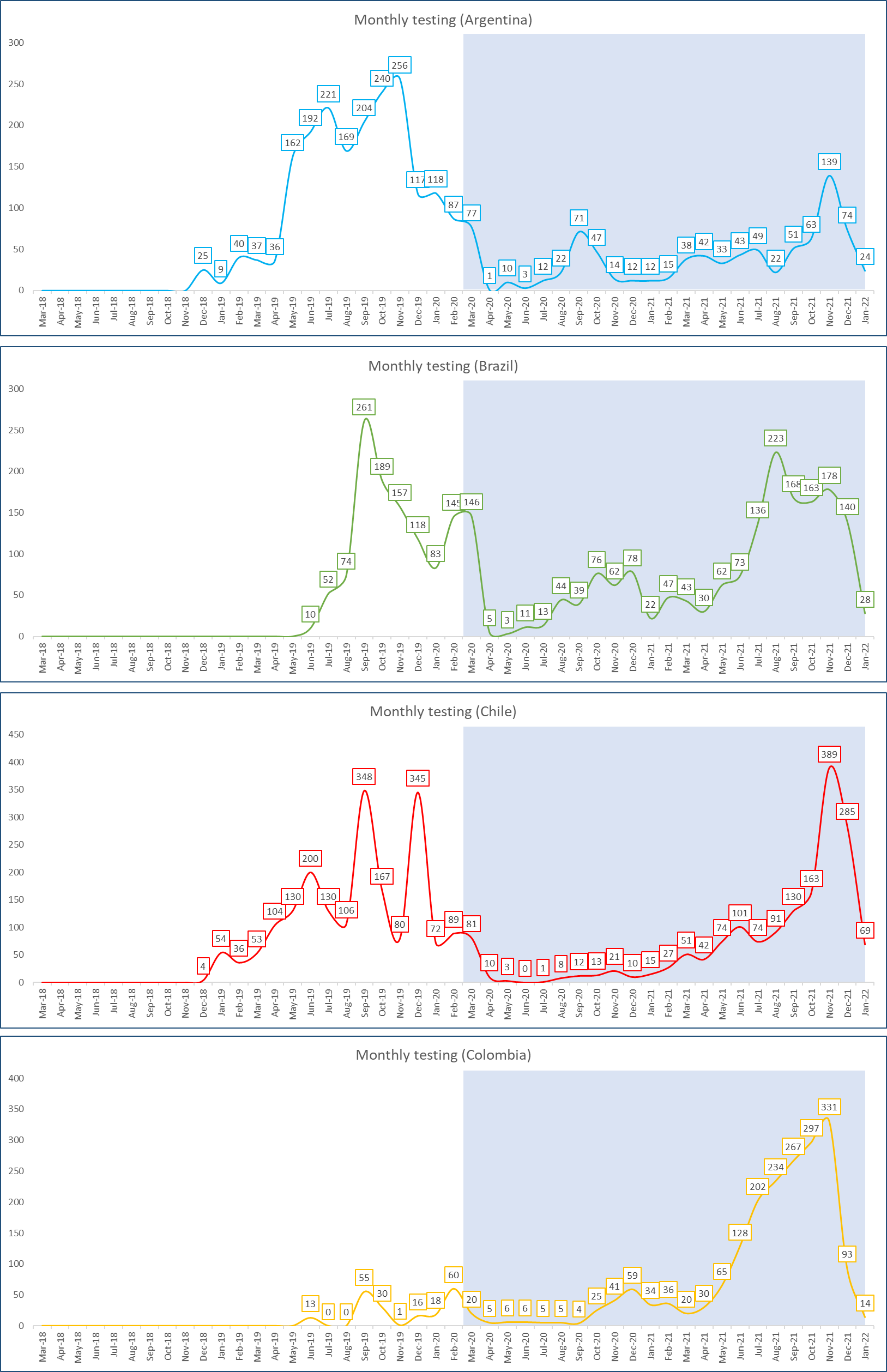


In light blue, effect of Coronavirus pandemic.

Figure 3S. Distribution of sample types by geographical area.


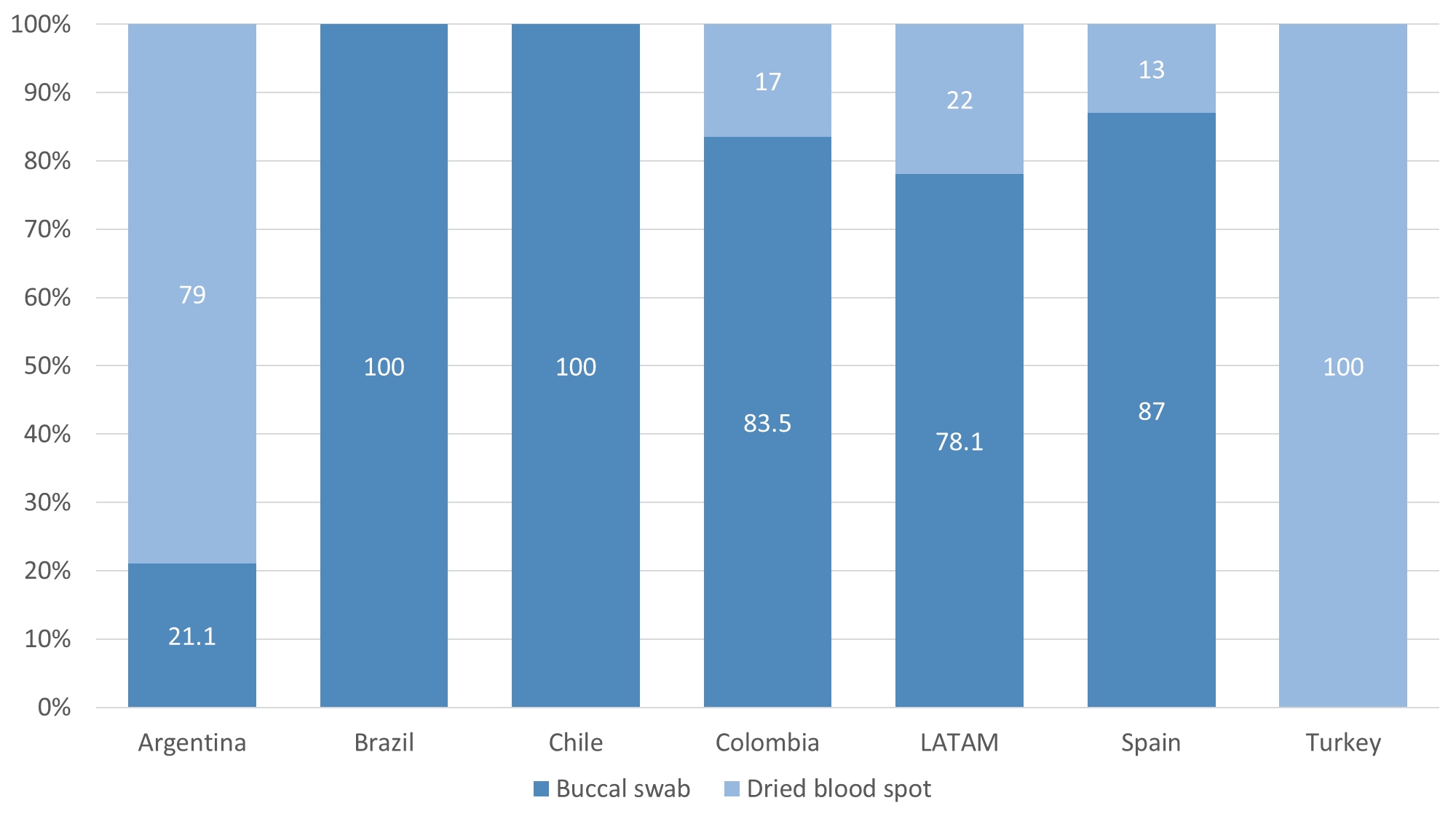


Figure 4S. Time elapsed between different steps by geographical area.


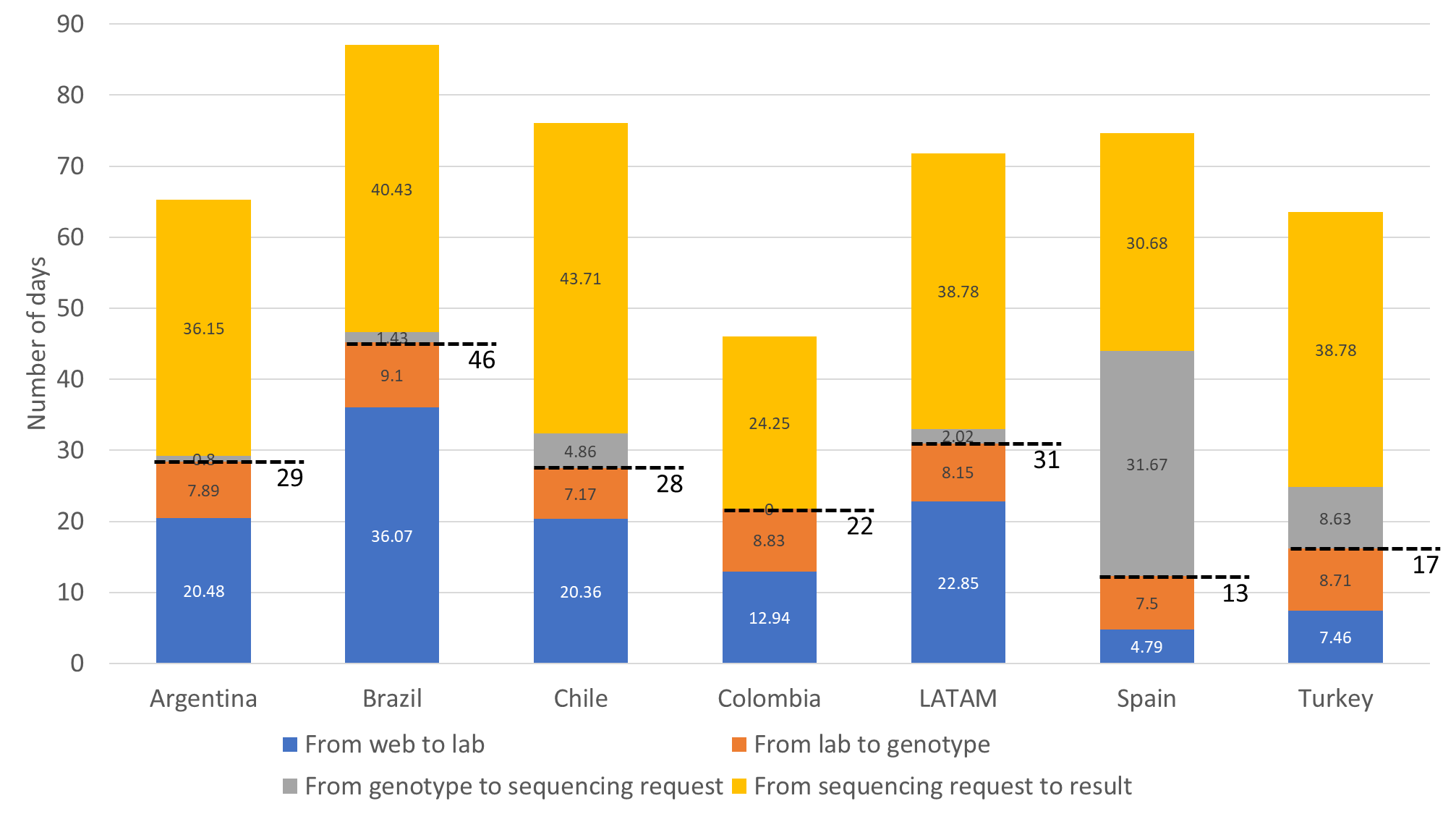


Figure 5S. Allele distribution according to the different genotyping reasons in different Latin American countries.


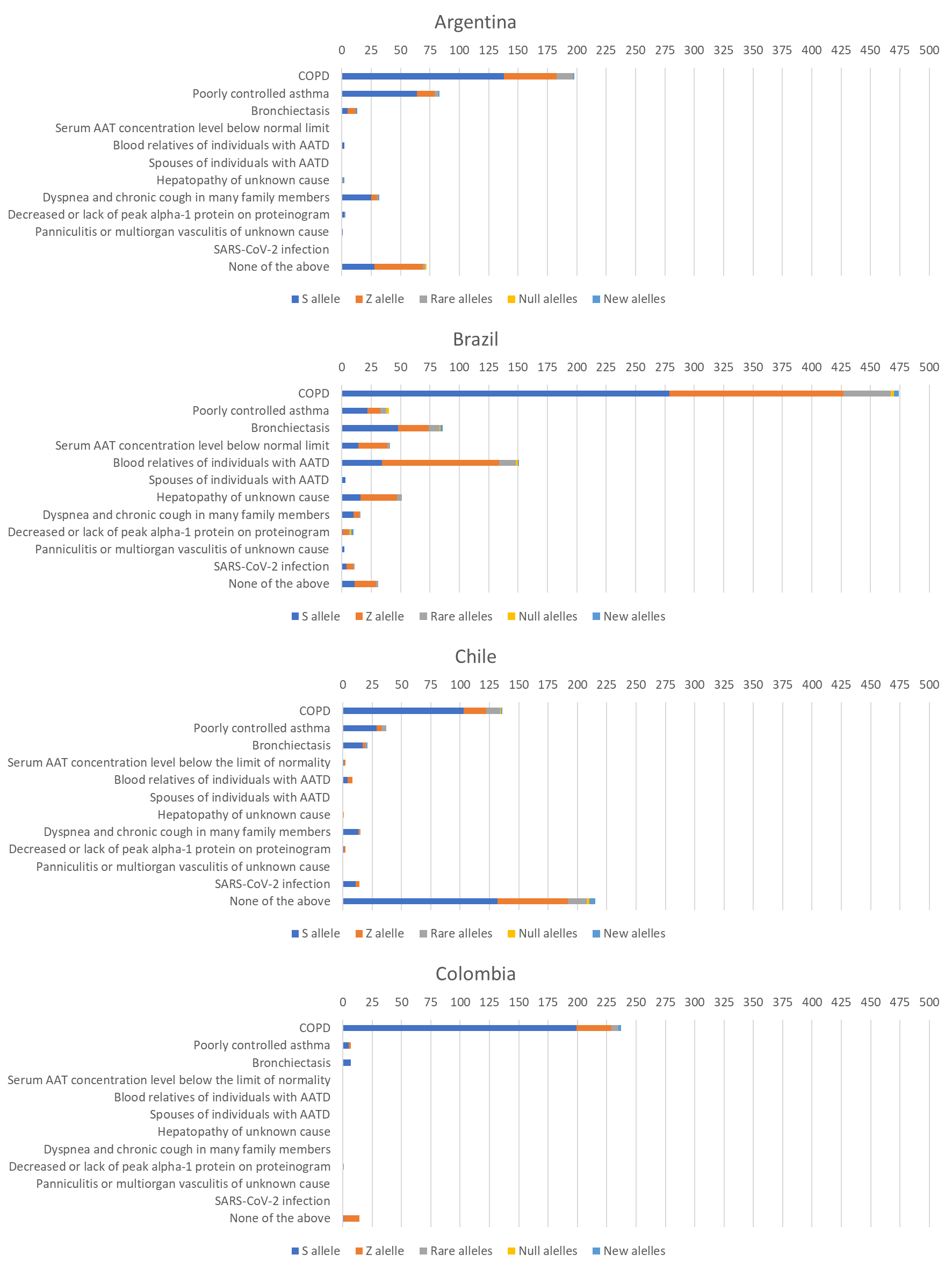


Table 1S. Description of the allelic variants and associated alleles tested by A1AT Genotyping Test; Information about the activity of the A1AT protein expressed

| **Allelic variant (RefSeq: NM_001127701.1)** | **Associated Alleles** | **Predicted Protein Activity** |
| --- | --- | --- |
| c.187C>T | **PI* I** | Reduced (mild) |
| c.194T>C | **PI* M procida** | Reduced (severe) |
| c.226_228delTTC | **PI* M malton**, PI* M palermo, PI* M nichinan | Reduced (severe) |
| c.230C>T | **PI* S iiyama** | Reduced (severe) |
| c.552delC | **PI* Q0 granite falls** | None (no protein) |
| c.646+1G>T | **PI* Q0 west** | None (no protein) |
| c.721A>T | **PI* Q0 bellingham** | None (no protein) |
| c.739C>T | **PI* F** | Reduced (mild) |
| c.839A>T | **PI* P lowell**, PI* P duarte, PI* Q0 cardiff, PI* Y barcelona | Reduced (severe) |
| c.863A>T | **PI* S**, PI* T | Reduced (mild) |
| c.1096G>A | **PI* Z**, PI* Z augsburg | Reduced (severe) |
| c.1130dupT | **PI* Q0 mattawa**, PI* Q0 ourem | None (no protein) |
| c.1158dupC | **PI* Q0 clayton**, PI* Q0 saarbruecken | None (no protein) |
| c.1178C>T | **PI* M heerlen** | Reduced (severe) |

Most frequent Associated Allele highlighted in bold

Table 2S. Cases in which sequencing revealed additional mutation from direct genotyping.

| Direct genotyping | Gene sequencing |
| --- | --- |
| -/Z | Z/Z-Wrexham |
| -/Z | Z/Z-Wrexham |
| -/Z | Z/M würzburg |
| -/Z | Z/M würzburg |
| -/Z | Z/M würzburg |
| Z/M malton | Z/M palermo |
| Z/M malton | Z/M palermo |
| Z/M malton | Z/M palermo |
| Z/Z | Z/c.1066-26C>T |
| -/Z | Z / c.1177C>A |
| -/S | S/Zbristol + c.-428G>A + c.-10T>C |
| -/S | S/Zbristol |
| -/S | S/QOmadrid |
| -/S | S/M würzburg |
| S/M malton | S/M palermo |
| -/- | Q0kayseri/Q0kayseri |
| -/- | Q0 Brescia/Q0 Brescia + c.-10T>C |
| -/P lowell | P lowell/Yorzinouvi |
| -/P lowell | P lowell / Z bristol |
| -/- | M/Zbristol |
| -/Z | M/Z + c.918-10T>A + c.917+37C>T |
| -/Z | M/Z + c.918-10T>A |
| -/Z | M/Z + c.287C>T |
| -/Z | M/Z + c.-428G>A + c.-10T>C |
| -/Z | M/Z + c.-428G>A + c.-10T>C |
| -/Z | M/Z + c.-428G>A + c.-10T>C |
| -/Z | M/Z + c.-428G>A |
| -/Z | M/Z + c.-428G>A |
| -/Z | M/Z + c.-344A>C |
| -/Z | M/Z + c.-10T>C |
| -/Z | M/Z + c.-10T>C |
| -/- | M/Z |
| -/S | M/S + c.97C>T |
| -/S | M/S + c.922G>T |
| -/S | M/S + c.922G>T |
| -/S | M/S + c.646+2T>G |
| -/S | M/S + c.485A>G |
| -/S | M/S + c.-428G>A |
| -/Q0 mattawa | M/Q0mattawa + c.1052del |
| -/- | M/Q0 madrid |
| -/P lowell | M/P lowell + Yorzinuovi |
| -/P lowell | M/P lowell + c.-109+41A>G |
| -/- | M/M würzburg |
| -/M procida | M/M procida + c.194T>C + c.853C>T |
| -/M procida | M/M procida + Q0porto |
| -/M malton | M/M palermo |
| -/M malton | M/M palermo |
| -/M malton | M/M malton + c.-428G>A + c.424C>T |
| -/- | M/M + c.871C>T |
| -/- | M/M + c.853C>T |
| -/- | M/M + c.647-69T>C + c.1033G>T |
| -/- | M/M + c.424C>T |
| -/- | M/M + c.286_287dup |
| -/- | M/M + c.286_287dup |
| -/- | M/M + c.211A>C + c.930del |
| -/- | M/M + c.1095C>G |
| -/- | M/M + c.1068C>T |
| -/- | M/M + c.1045G>C |
| -/- | M/M + c.1033G>T |
| -/- | M/M + c.-5+1G>A + c.-428G>A + c.-10T>C |
| -/- | M/M + c.-428G>A HOM+ c.424C>T HOM |
| -/- | M/M + c.-428G>A + c.424C>T |
| -/- | M/M + c.-428G>A + c.424C>T |
| -/- | M/M + c.-428G>A + c.424C>T |
| -/- | M/M + c.-428G>A + c.424C>T |
| -/- | M/M + c.-428G>A + c.-342G>A + c.424C>T + c.647-71T>C |
| -/- | M/M + c.-428G>A + c.-127G>A + c.-10T>C |
| -/- | M/M + c.-428G>A + c.-10T>C + c.109+41A>G |
| -/- | M/M + c.-428G>A + c.-10T>C + c.109+41A>G |
| -/- | M/M + c.-428G>A + c.-10T>C |
| -/- | M/M + c.-428G>A + c.-10T>C |
| -/- | M/M + c.-428G>A + c.-10T>C |
| -/- | M/M + c.-428G>A + c.-10T>C |
| -/- | M/M + c.-428G>A + c.-10T>C |
| -/- | M/M + c.-428G>A + c.-10T>C |
| -/- | M/M + c.-428G>A |
| -/- | M/M + c.-428G>A |
| -/- | M/M + c.-428G>A |
| -/- | M/M + c.-428G>A |
| -/- | M/M + c.-428G>A |
| -/- | M/M + c.-428G>A |
| -/- | M/M + c.-428G>A |
| -/- | M/M + c.-342G>A + c.647-71T>C |
| -/- | M/M + c.-10T>C + c.-109+56C>T |
| -/- | M/M + c.-10T>C |
| -/- | M/M + c.-10T>C |
| -/- | M/M + c.-10T>C |
| -/- | M/M + c.-10T>C |
| -/- | M/M + c.-10T>C |
| -/- | M/M + c.-109+56C>T |
| -/- | M/M + c-10T>C + c.424C>T + c.725_727dup |
| -/- | M + c.1066-26C>T |
| -/- | M + c.1066-26C>T |
| -/- | c.286_287dup HOM |
